# CuO-Decorated ZnO Hierarchical Nanostructures as Efficient and Established Sensing Materials for H_2_S Gas Sensors

**DOI:** 10.1038/srep26736

**Published:** 2016-05-27

**Authors:** Nguyen Minh Vuong, Nguyen Duc Chinh, Bui The Huy, Yong-Ill Lee

**Affiliations:** 1 Department of Chemistry, Changwon National University, Changwon 641-773, Republic of Korea; 2 Department of Physics, Quy Nhon University, 170 An Duong Vuong, Quy Nhon, Binh Dinh, Vietnam; 3 Department of Materials Science and Engineering, Chungnam National University, Daejeon, 305-764, Republic of Korea

## Abstract

Highly sensitive hydrogen sulfide (H_2_S) gas sensors were developed from CuO-decorated ZnO semiconducting hierarchical nanostructures. The ZnO hierarchical nanostructure was fabricated by an electrospinning method following hydrothermal and heat treatment. CuO decoration of ZnO hierarchical structures was carried out by a wet method. The H_2_S gas-sensing properties were examined at different working temperatures using various quantities of CuO as the variable. CuO decoration of the ZnO hierarchical structure was observed to promote sensitivity for H_2_S gas higher than 30 times at low working temperature (200 °C) compared with that in the nondecorated hierarchical structure. The sensing mechanism of the hybrid sensor structure is also discussed. The morphology and characteristics of the samples were examined by scanning electron microscopy (SEM), X-ray diffraction (XRD), X-ray photoelectron spectroscopy (XPS), UV-vis absorption, photoluminescence (PL), and electrical measurements.

Hydrogen sulfide (H_2_S) is a colorless gas with a characteristic foul odor of rotten eggs. It mainly originates from the anaerobic digestion process due to the bacterial breakdown of organic compounds in the absence of oxygen gas such as in a swamp and in sewers. It occurs naturally in certain food, natural gas, and crude petroleum and also as industrial byproducts^1^,^2^. H_2_S is one of the most toxic gases and poses a health risk at high concentrations as apart from its unpleasant smell even at relatively low concentration^3^. In addition, H_2_S is corrosive, flammable, and explosive. The lower explosive limit of H_2_S for flammability is approximately 4%^4^, however, sensors for flammability alarms as well as health risk sensors require the reliable detection of trace concentrations in the environment.

To date, several types of sensor, such as chemoresistor sensors based on a semiconducting metal oxide, electrochemical sensors, optical sensors, conducting polymeric sensors, and piezeoelectric sensors, have been utilized for H_2_S gas detection^1^. However, metal oxide-based sensors show many advantages because of their small size, low power consumption, simple construction, low weight, and low cost. Therefore, they have been the most commonly used sensors for H_2_S gas monitoring. Metal oxide semiconductor sensor structures were produced with high porosity, large surface area, a larger number of active sites, and high surface catalytic activation with the aim of improving the gas-sensing performance^5^,^6^,^7^. Among the investigated metal oxide sensing materials, the n-type ZnO semiconductor has been extensively researched because of its advantageous properties such as high electron mobility and good chemical/thermal stability.

Recently, ZnO nanostructure-based sensor performance was significantly improved by the hybridization of ZnO with other components such as carbon materials, noble metals, and metal oxides^8^,^9^,^10^,^11^,^12^. Sensors based on multi-components are more sensitive than the pure ZnO material suggesting the combination of many different characteristics such as a change in conductance, improved surface catalytic property, increasing surface reaction sites, and high porosity. In addition, the formation of a contact potential at the interface between components also contributes to the improvement of the gas-sensing performance. The contact potential is sensitive to the type of ambient gas; hence, the resistance of the hetero-contact is greatly influenced by the ambient gas^13^. Copper (II) oxide (CuO) material, a p-type semiconductor because of the existence of Cu vacancies, has been recognized as an important catalyst on ZnO surface for the improvement of H_2_S gas-sensing performance. The conversion of CuO into copper sulfide (CuS) upon exposure to H_2_S gas was considered to be the main reason for the large change in surface conductance and thus the sensing property^11^,^12^,^14^. However, the sensor read-out of the previous CuO/ZnO composite structures still remains some limitations including high power consumption^11^,^15^, low sensitivity^11^,^12^,^14^,^16^,^17^ as well as limited selectivity^11^,^12^. In addition, the reproducibility and satisfactory consistency of sensors have not studied systematically. Therefore, the exploration of new structure of hierarchical ZnO/CuO as a gas sensor is desired as a challenging task to achieve higher sensitivity towards H_2_S at lower working temperature with good reproducibility and selectivity.

Here we report a facile strategy for the preparation of an open space CuO-decorated ZnO hierarchical nanostructure. The first essential feature our sensor structure required was a ZnO hierarchical (ZnO-H) structure with relatively large open spaces using ZnO nanofibers (ZnO-Fs) as a template for ZnO nanorod growth such that the gases could freely flow and maintain contact with the entire effective surface of the sensing material with minimal diffusion effect. The second essential feature is controllable uniform decoration of the ZnO surface with CuO nanoparticles. Herein, we systematically examined the effects of various amounts of CuO nanoparticles on the structural, optical, electrical, and H_2_S gas-sensing properties of the fabricated open space hierarchical sensor. A great improvement in sensitivity towards H_2_S gas at a low working temperature was observed. The adsorption-desorption kinetic processes on the surface of the CuO nanoparticles and the ZnO-H nanostructure are also discussed.

## Results

### Morphology and structural properties

**Figure 1:**
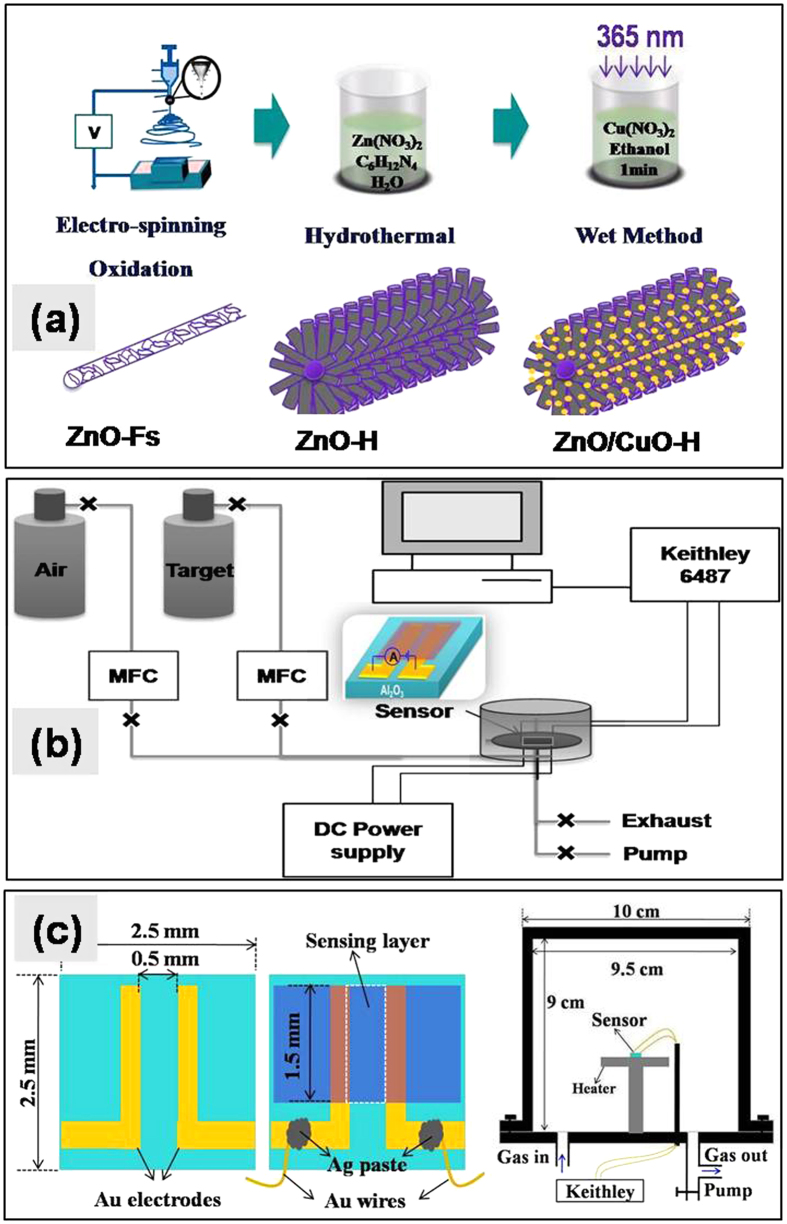
(**a**) Flowchart of the fabricated process for open-space porous CuO-decorated ZnO hierarchical structure. (**b**) Schematic diagram of the gas-sensing apparatus and (**c**) schematic illustration of gas sensor structure and reactor chamber.

**Figure 2:**
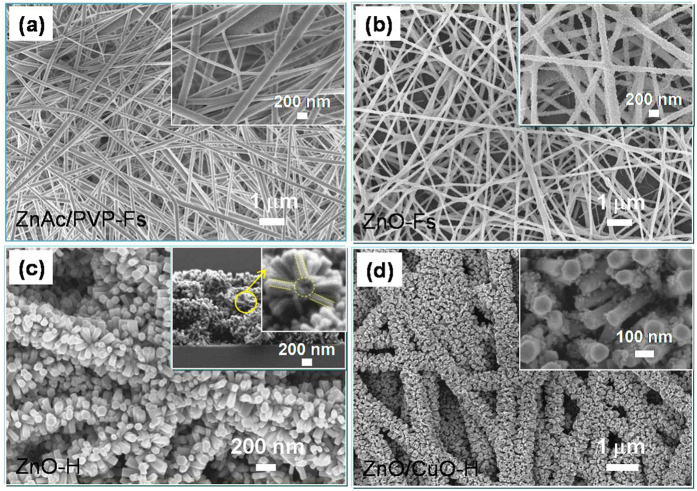
Morphology of the (**a**) ZnAc-PVP nanofibers, (**b**) ZnO-Fs formed by oxidation of (**a**) at 500 °C, (**c**) ZnO-H structure fabricated by hydrothermal method using ZnO-Fs in (**b**) as seed template, and (**d**) ZnO/CuO-H structure formed by decoration of CuO nanoparticles on structure in (**c**). Inset images in (**a,b,d**) show high magnification SEM. Inset image in (**c**) shows cross-section view.

Figure 1 shows the flowchart of the fabrication process for open space porous CuO decorated ZnO-H nanostructures (Fig. 1a), a schematic diagram of the gas-sensing apparatus (Fig. 1b), and a schematic illustration of the structure of the gas sensor as well as the reactor chamber (Fig. 1c). Herein, Au patterned Al_2_O_3_ was utilized as sensor substrate. Figure 2(a–d) show the SEM morphologies of the ZnAc-PVP composite nanofibers, ZnO-Fs, ZnO-H, and ZnO/CuO hierarchical (ZnO/CuO-H) structures, respectively. The insets in (Fig. 2a,b,d) and (Fig. 2c) show the high-magnification and cross-section SEM images, respectively. The ZnAc-PVP composite nanofibers (Fig. 2a) with diameters between 100 nm and 250 nm appear to have relatively smooth surfaces because of the polymeric property and/or amorphous nature of ZnAc-PVP^18^,^19^. During oxidation, the average diameter of ZnO-Fs shrinks slightly to diameters between 60 and 200 nm (Fig. 2b) and this shrinkage is attributable to the crystallization of ZnO as well as the PVP having burnt out of the nanofibers. The random and uniform distribution of large (hundreds of nanometers) spaces between nanofibers was not changed by oxidation. As shown in Fig. 2b, ZnO-Fs were formed by embedding ZnO nanoparticles with average grain sizes from 20 to 40 nm (Fig. 2b-inset). These ZnO nanoparticles on the nanofibers are useful as a seed template for the growth of ZnO nanorods in the next step.

Figure 2c shows an SEM image of the ZnO-H structures obtained by hydrothermal growth of ZnO nanorods using polycrystalline ZnO-Fs as a seed template. The average thickness of the ZnO-H film is approximately 1.1 μm (Fig. 2c inset). The SEM images clearly show that the secondary ZnO nanorods resulting from hydrothermal growth are organized into very regular arrays formed symmetrically around the ZnO-Fs (known as a hierarchical structure Fig. 2c inset). The open spaces in the ZnO-H structure were expected to achieve a high gas-sensing performance because they enable gases to freely flow and make contact with the entire ZnO surface with minimal diffusion effect^5^. Figure 2d shows an SEM image of the ZnO-H structure decorated with CuO nanoparticles, which accumulated on the ZnO nanorods after dip-coating treatment in a copper salt solution using UV illumination following the oxidation step. The CuO coating changed the surface roughness of ZnO to a scale of tens of nanometers (Fig. 2d inset).

**Figure 3:**
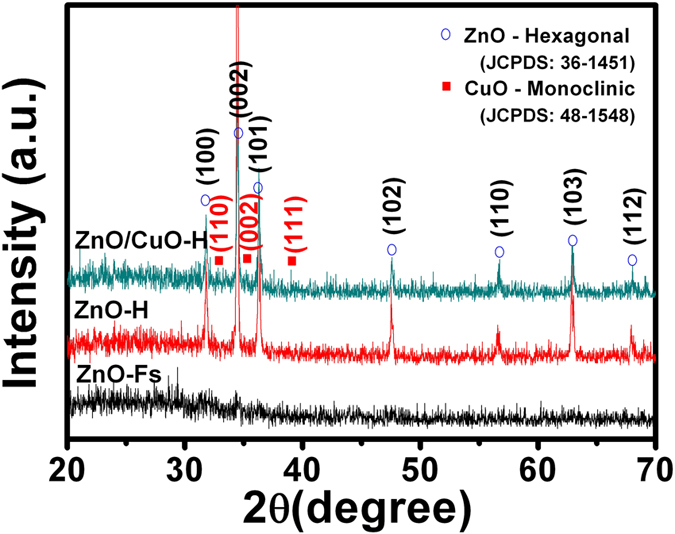
XRD patterns of ZnO-Fs, ZnO-H, and ZnO/CuO-H (5 mM) structures.

The XRD patterns of the ZnO-Fs, ZnO-H, and ZnO/CuO-H structures on glass substrates are compared in Fig. 3. Note that the ZnO-Fs, ZnO-H, and ZnO/CuO-H structures showed similar morphologies on both the Al2O3 and glass substrate as shown in Fig. S1a. All of them exhibited a hexagonal wurtzite ZnO structure with lattice parameters of a = 3.25 Å and c = 5.21 Å [JCPDS 36-1451] and high crystallinity except for the ZnO-Fs of which the diffraction peaks were not identified due to the sparse amount of ZnO-Fs on the glass substrate. The strong diffraction peak in the hierarchical structures centered at a scattering angle of 34.5° for the (002) diffraction plane of the wurtzite type of ZnO, dominates the other peaks, and provides evidence that the growth process of ZnO nanorods is highly oriented in the 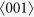 direction on the ZnO-Fs. The result also showed a monoclinic CuO structure with its main peaks at (110), (002), and (111) [JCPDS file no. 48-1548] for the ZnO/CuO-H nanostructure. However, these diffraction peaks are quite weak, which is attributed to the very small amount of CuO decoration required to obtain strong diffraction peaks.

**Figure 4:**
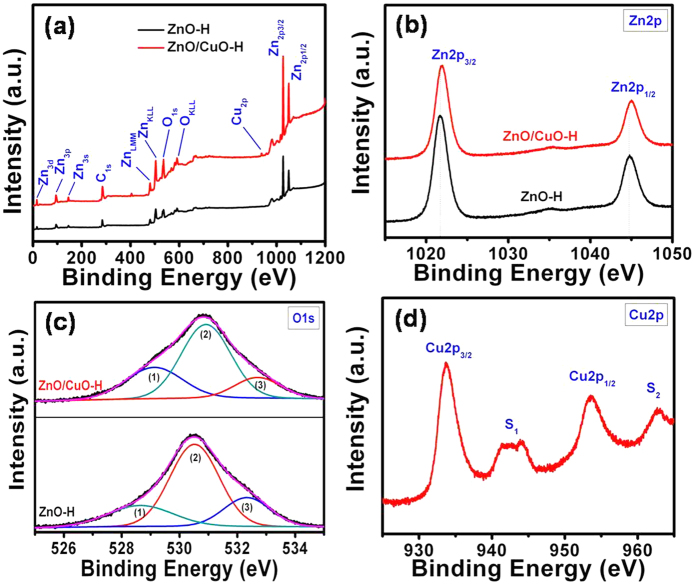
(**a**) XPS spectrum of pure ZnO-H, and ZnO/CuO-H (5 mM) structures. (**b–d**) High-resolution spectrum of Zn, O, and Cu peaks, respectively.

The surface composition and chemical states of the elements existing in the sample were investigated by recording XPS survey scans of the ZnO-H, and ZnO/CuO-H structures as shown in Fig. 4a. The result indicated the presence of the elements Zn, O, and C in the samples and, additionally, the element Cu in the ZnO/CuO-H structure. The peak at 285.35 eV is attributed to the CO_2_ commonly adsorbed on the surface of the sample^20^ and/or carbon that remained in small quantities after burning out the PVP at 500 °C. Comparison of the Zn2p peaks of the pure ZnO-H and ZnO/CuO-H samples is presented in Fig. 4b. The Zn2p peaks centered at 1021.65 and 1044.81 eV (for the ZnO-H structure) are assigned to the Zn2p_3/2_ and Zn2p_1/2_ levels, respectively^21^. These peaks correspond to Zn^2+^ in a hexagonal wurtzite ZnO structure. The Zn2p peaks are seen to be shifted to slightly higher binding energies for the ZnO/CuO-H sample of 1021.96 eV (Zn2p_3/2_) and 1045.04 eV (Zn2p_1/2_). The high-resolution O1s peak (Fig. 4c) exhibited multiple overlapping components. This peak was fitted with typical Gaussian functions and resolved to peaks (1), (2), and (3) with binding energies of 528.64, 530.50, and 532.32 eV for the ZnO-H structure, respectively. Peak (2) may be related to O^2−^ species in the lattice^21^. Peak (1) may be attributed to O_2_^−^ ions adsorbed on the surface of the film^22^, whereas peak (3) may have been caused by the ions of O_2_^2−^, O^−^, and OH^−^ in oxygen-deficient regions^23^,^24^. In the case of the ZnO/CuO-H sample, the O1s peaks from (1) to (3) are observed to be shifted to slightly higher binding energies of 529.15 eV, 530.90 eV, and 532.73 eV, respectively. Briefly, the shift to higher binding energies in the ZnO/CuO-H sample compared to the ZnO-H structure occurred for both the Zn2p and O1s levels. The shift in the binding energies of Zn2p can be ascribed mainly to the interaction between CuO nanoparticles and ZnO material, whereas that of O1s clearly depicted the changes in the oxygen environment at the surface due to CuO coating. The changes in the surface oxygen species in the samples were additionally confirmed by calculating the ratio of the integrated areas of (peak (1) + peak (3)) and peak (2). These ratios are 0.67 and 0.75 for the ZnO-H and ZnO/CuO-H samples, respectively, indicating an increase in the amount of absorbed oxygen species in the ZnO/CuO-H structure compared to the pure ZnO-H structure. However, this increment is not the main reason for the improvement in H_2_S gas-sensing performance discussed below. The high-resolution Cu2p spectra are shown in Fig. 4d. The peak at 933.63 eV is attributed to Cu2p_3/2_, whereas the peak at 953.61 eV is ascribed to Cu2p_1/2_, indicating the existence of CuO nanoparticles with the +2 oxidation state of Cu^21^. In addition, satellite peaks of Cu2p_3/2_ and Cu2p_1/2_ were observed as peaks (S1) and (S2), respectively, characteristic of a partially filled d-orbital (3d^9^ in the case of Cu^2+^)^25^. The XRD and XPS results strongly support the formation of CuO with Cu (II) on the ZnO surface.

### Optical properties

**Figure 5:**
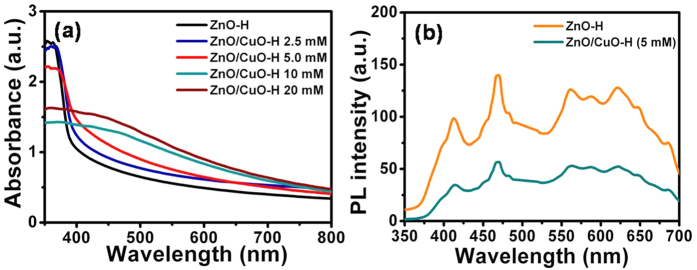
(**a**) UV-visible absorption and (**b**) photoluminescence spectra of pure ZnO-H and ZnO/CuO-H (5 mM) structures.

The optical properties of pure ZnO-H and ZnO/CuO-H structures were characterized by UV-vis absorption (Fig. 5a) and PL (Fig. 5b). The absorption spectrum was recorded by growing hierarchical structures on a glass substrate instead of on Al_2_O_3_ and a blank glass substrate was used as reference. Figure 5a shows the absorption spectra of hierarchical structures of ZnO and ZnO decorated with CuO at different concentrations. The absorption edge of the films is determined from the intersection of the sharply decreasing region of the spectrum and its baseline^26^. The spectra of ZnO-H show a band gap absorbed edge at around 398 nm that results from the electron transition from the valence band to the conduction band. The absorptivity in the UV region is due to both absorption and scattering, whereas that in the visible light region is consistent with scattering by nanorods with a small diameter only. Importantly, a gradual red shift in the absorption edge values was observed as the amount of CuO coating increased. The presence of low amounts of CuO with concentrations of 2.5 and 5 mM shows enhanced absorbance of visible light, which can be attributed to the localized energy states in the band gap of ZnO due to the formation of defects in the ZnO lattice during CuO decoration and/or enhanced light-scattering effects. Moreover, these samples also exhibit a slight shift to a longer wavelength in the absorption edges of 405 nm (at 2.5 mM of CuO) and 422 nm (5 mM of CuO). The red shift in the band edge of ZnO/CuO-H structure is similar to that observed in previous research^25^. A significant red shift in the absorption edge is obtained for samples with higher CuO concentrations (750 nm at 10 mM CuO and 765 nm at 20 mM). This behavior is probably due to the incorporation of excessive amounts of CuO nanoparticles that have a small band gap value (1.5 eV).

Figure 5b shows a comparison of the room-temperature PL spectra of pure ZnO-H and ZnO/CuO-H (5 mM) structures under an excitation wavelength of 325 nm. It shows clearly that the PL intensity of the ZnO/CuO-H structure is lower than that of the pure ZnO structure indicating that the formation of p-CuO/n-ZnO junctions are evident of the suppression of electron–hole recombination. The shape of the PL spectra remained unchanged for both samples. Emission bands are obtained in the ultraviolet (UV) and visible regions (orange, yellow, green, blue and violet) as shown in Fig. S1b after Gaussian fitting. It is well known that there are six types of point defects in the ZnO lattice. Among them, the donor defects are V_o_, Zn_i_, and Zn_O_, whereas the acceptor defects are V_Zn_, O_i_, and O_Zn_. The ultraviolet emission shoulder (392 nm) is usually considered as the characteristic emission of ZnO and is closely related to the band – band transition and/or the exciton recombination^27^. The violet (412 nm) and blue emissions (452 and 469 nm) are attributed to the transitions from the Zn_i_ and extended Zn_i_ states to the valence band, respectively^28^. The green emissions (514, 548, and 562 nm) involve transitions from the shallow donor and conduction band to the deep acceptor levels, respectively^28^,^29^. The origin of the visible emission peaks centered at ~586–713 nm is generally attributed to the transitions from the Zn_i_ states or the conduction band to the deep levels (V_o_ and O_i_)^30^,^31^.

### DC conductivity measurements

**Figure 6:**
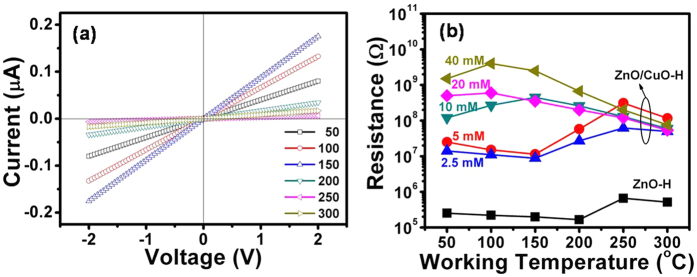
(**a**)Current – voltage characteristic of ZnO/CuO-H (5 mM) structure sensor in dry ambient air, (**b**) Dependence of the resistance of the pure ZnO and CuO-decorated ZnO-H structures on different working temperatures.

The current–voltage (I-V) characteristics of the fabricated hierarchical devices, as fundamental properties of electronic materials, were measured by varying the temperature in dry air. Prior to the I-V measurements, the devices were heated to 350 °C under the given ambient conditions to remove the effect of adsorbed water molecules on the oxide surface. The I-V curves of hierarchical structures of pure ZnO (not shown here) and ZnO/CuO (Fig. 6a) are linear, indicating the formation of Ohmic contacts between the hierarchical structure and Au electrodes. The resistance of sensors calculated from slope the I-V curves for pure ZnO-H and ZnO/CuO-H structures are shown in Fig. 6b.

It is shown that the resistance of the ZnO/CuO-H sensors is approximately two orders of magnitude higher than that of a pure ZnO-H sensor. The higher resistance of the ZnO sensor after CuO decoration suggests that the formation of a p-n junction (at the interface between the n-type ZnO and p-type CuO particles) depleted electrons from the ZnO layer more effectively than the surface oxygen adsorption. Notably, the pure ZnO-H, ZnO/CuO-H (2.5 mM), and ZnO/CuO-H (5 mM) samples revealed a monotonic decrease in resistance with increasing temperature in the low-temperature measuring region (50–150 °C). This relationship indicates dominant semiconducting behavior in this temperature region. Therefore, the semiconducting nature of CuO did not alter the semiconducting resistance–temperature behavior of the ZnO probably because of the discreteness of the distribution of CuO particles on the surface (Fig. 2d-inset).

Interestingly, the temperature-dependent resistance behavior of the pure ZnO-H, ZnO/CuO-H (2.5 mM), and ZnO/CuO-H (5 mM) sensors deviated from the semiconducting behavior in the high-temperature region (150–300 °C); the resistance of these materials increased with increasing temperature. A “local maximum point,” which does not determine the sensitivity of the sensor, is found in the sensor resistance at 250 °C. However, the sensitivity of the sensor is expected to improve in the vicinity of this point. The increase in resistance is attributed to an enhanced oxygen ionosorption rate and corresponding increase in the surface depletion depth of the ZnO nanostructure. Moreover, we also observed a further increase in the resistance of the ZnO/CuO-H (2.5 and 5 mM) sensors compared to the pure ZnO-H sensor. This phenomenon is attributed to the catalytic effect of the embedded CuO nanoparticles on the ZnO surface in an environment containing air. We found the catalytic effect of CuO to critically enhance the dissociation of oxygen molecules at >150 °C, at which the ionic adsorption form of oxygen changes from O_2_^−^ to O^− ^32.

A significant increase in sensor resistance is obtained in the temperature region (50–200 °C) when the content of embedded CuO nanoparticles (10, 20 and 40 mM) is increased even further, and this is attributed to the formation of a large number of p-n heterojunctions on the ZnO surface. Moreover, the presence of CuO nanoparticles on the ZnO surface may increase the number of gas absorption sites. However, if these sites are too densely packed, the decorated CuO nanoparticles may prevent the gas from coming in contact with the ZnO surface. The “local maximum point” in sensor resistance is obtained around 150 °C and 100 °C for the ZnO-H sensors decorated with CuO concentrations of 10 mM and 20 or 40 mM, respectively. This suggests that the oxygen absorbed on CuO is dominant compared with that on the host ZnO semiconductor in the low-temperature working region. We subsequently sought further confirmation of this conclusion by investigating the electrical and gas-sensing properties (discussed in the next section) of the pure CuO sensor. Herein, because the wet method to prepare this sensor produces the CuO product in very low yield, the porous CuO nanowire structure sensor (Fig. S2a,b) was fabricated by Cu metal deposition on a single-wall carbon nanotubes template following oxidation at 800 °C with different deposition times of 8 and 2 min, as previously reported by our group^5^,^33^. The average diameters of the CuO nanowires are around 125 nm and 40 nm. CuO nanowires show a monoclinic structure as indicated by the XRD pattern (Fig. S2c). An increase was observed in both the CuO sensor resistance and H_2_S gas sensitivity with decreasing working temperature (Fig. S3a), thereby indicating semiconductor behavior of CuO, as well as the dominance of oxygen absorption on the CuO surface in the low-temperature working region.

### Gas-sensing properties

**Figure 7:**
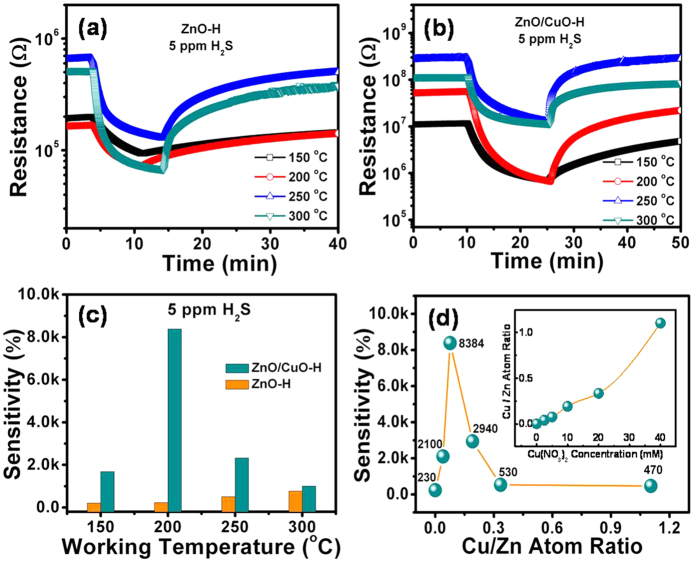
Gas-sensing properties of pure ZnO-H (**a**) and ZnO/CuO-H (5 mM) (**b**) structure sensors upon exposure to 5 ppm H_2_S at different working temperatures. (**c**) The sensitivity of sensors to 5 ppm H_2_S at different working temperatures are summarized. (**d**) Dependence of the CuO decorated ZnO sensor sensitivity on different CuO concentrations. Inset image show dependence of Cu/Zn atom ratio calculated by XPS spectra on different Cu salt concentrations.

The effect of the working temperature on the H_2_S gas-sensing properties was examined for pure ZnO-H and ZnO/CuO-H (5 mM) samples. Measurements were not obtained for the sensor based on ZnO-Fs because of its extremely high resistance at all working temperatures, which is out of the range of our instrument. Figure7a,b show the response behavior upon exposure to 5 ppm H_2_S diluted in dry air at different working temperatures (150–300 °C) of sensors based on ZnO-H and ZnO/CuO-H (5 mM) structures, respectively. According to previous reports, the catalytic decomposition of H_2_S occurs at high temperatures (>300 °C)^33^,^34^,^35^ causing the formation of a shallow donor level in the band gap due to the diffusion of sulfur in ZnO^36^. The species binding on the sensing layer changes at ~300 °C. Therefore, the working temperature was measured below 300 °C for the sensing of H_2_S gas to distinguish it from the different sensing mechanisms above 300 °C. The sensitivity of the sensor was defined by the R_i_/R_f_ ratio, where R_i_ is the baseline resistance of the sensor in ambient air and R_f_ is the resistance upon exposure to reducing gas at a given temperature. Both of these sensors exhibited gas-sensing behavior typical of an *n*-type semiconductor because the base resistance of the sensor decreased with exposure to the H_2_S reducing gas. The observed sensitivity measurements and their dependence on the working temperatures are summarized in Fig. 7c. The following observations were made: (1) the sensitivity of the ZnO-H sensor increases with increasing working temperature, whereas the ZnO/CuO-H sensor shows an optimal working temperature of 200 °C. (2) a remarkable improvement in the sensitivity resulting from CuO decoration was observed at all the measured working temperatures. The highest sensitivity of the ZnO-H and ZnO/CuO-H sensors to 5 ppm H_2_S were 767% and 8384% at the optimal working temperatures of 300 and 200 °C, respectively. This result can definitely be explained by the catalytic effect of the CuO nanoparticles. The performance of the ZnO/CuO-H sensor is comparable with or higher than that of the recently developed sensors based on the ZnO/CuO structure, as reviewed in Table 1. The enhanced H_2_S gas sensitivity as a result of the ZnO/CuO-H structure compared with other research can be attributed to the unique open-space porous ZnO-H structure. This hierarchical structure facilitates loading the CuO nanoparticles more evenly, resulting in the formation of efficient p-n heterojunctions between the CuO nanoparticles and ZnO materials, which in turn significantly affects the H_2_S gas-sensing performance, as discussed in the next section.

Previously, Tepore *et al.*^37^ performed a systematic examination to show that the response and recovery processes of the conductive sensing material towards reducing gases are the results of thermally activated chemical reaction processes on the gas-sensing surface. The gas-sensing behavior of oxide semiconductors has been explained by the ionosorption model, combined with the semiconductor junction theory. In detail, oxygen molecules in the dry ambient air absorb continuously on the empty absorption sites on the sensing surface at a given working temperature^32^ via


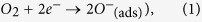


resulting in an electron depletion layer near the surface. When the ZnO material is exposed to H_2_S, the H_2_S gas molecules react continuously with the pre-absorbed oxygen ions (O^−^) via


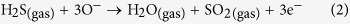


to form both H_2_O and SO_2_ in gaseous form. This reduction of oxygen ions on the ZnO surface thins the depletion layer because electrons are released into the ZnO material, thereby causing the conductivity to increase. In this work, the sensing mechanism was further examined by analyzing the response and recovery rates of the sensor. Herein, the response and recovery times were measured assuming the exponential rise and decay of the curves based on the first-order surface reaction kinetics for adsorption and desorption as shown in our previous reports^37^,^38^,^39^,^40^. The changes in conductance are expressed^40^ by


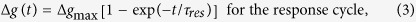



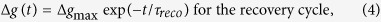


where, Δ*g*, Δ*g*_*max*_*, τ*_*res*_, and *τ*_*reco*_ are the time-dependent conductance change, maximum conductance change, response, and recovery times, respectively. Therefore, the response and recovery times are the characteristic average times of the processes and are the times required for completion of approximately 63% of the response and recovery processes. Figure S4a,b show the plots redrawn from the response and recovery cycles of Fig. 7a according to Eqs (3) and (4) at various working temperatures, respectively. The response and recovery times at each temperature can be estimated from the slopes in Fig. S4a,b, respectively, and are summarized in Fig. S5. The results revealed decreasing response and recovery times with increasing working temperature. The response times of ~500, ~238, ~204, and ~166 s, and recovery times of ~1502, ~746, ~233, and 116 s were obtained at the working temperatures of 150, 200, 250, and 300 °C, respectively. This observation indicates the promoting reaction rate, which is governed by the rates of H_2_S gas decomposition and/or surface reaction, of H_2_O and SO_2_ formation in Eq. (2) in response process^41^. At the same time, it is necessary to enhance desorption of H_2_O and SO_2_ molecules and/or enhance oxygen decomposition followed by adsorption by increasing the working temperature.

Nevertheless, the slopes of the recovery curves in Fig. S4b differ. The slope of the recovery curve at 150 °C is constant, whereas the slopes at higher working temperatures (200, 250, and 300 °C) exhibit a transition. This observed change in the slopes during the recovery cycle reveals a complex process occurring on the sensing surface, namely a fast process in the beginning, followed by slow processes. Therefore, processes other than the formation of H_2_O and SO_2_ by Eq. (2) may have to be concluded for the sulfuration and desulfuration reactions. The sulfuration involving the transformation of ZnO to ZnS^42^ via


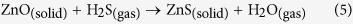


was also proposed to be the reason for the H_2_S response. Herein, the ZnS spots generated at the surface act as shield layer to effectively depress the extraction of free electrons from ZnO caused by oxygen absorption. Therefore, it is suggested that the fast process at the beginning of the response and recovery of ZnO and ZnO/CuO may reflect the rapid re-adsorption of oxygen onto the surface as shown in Eq. (1), whereas the subsequent slow processes might reflect the de-sulfuration for the transformation of ZnS to ZnO^42^ via


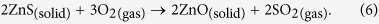


It was reported that, the change in the Gibbs free energy (Δ*rG*^o^) for the sulfuration reaction of Eq. (5) is −74.08 kJ/mol (at 25 °C) and −68.194 kJ/mol (at 150 °C), and that for the desulfuration of Eq. (6) is −838.72 kJ/mol (at 25 °C) and −820.35 kJ/mol (at 150 °C)^42^. The calculated thermodynamic data indicate that both of these reactions can spontaneously occur and are favored at low temperature. This can explain the ability of ZnO material to respond to H_2_S at low temperature as previously reported^43^,^44^. Moreover, the desulfuration process of ZnS oxidation tends to occur rapidly because the value of Δ*rG*^o^ is more negative. Therefore, ZnS formation simply is an intermediate process leading to a metastable temporary product in the H_2_S gas-sensing mechanism. This mechanism based on the surface reaction seems to be more dominant than that based on the sulfuration-desulfuration model at a higher working temperature because of the promotion of surface reactions resulting from the short response and recovery times (Fig. S5) as well as an increase in the sensitivity of the sensor as the working temperature increases (Fig. 7c). In fact, a combination of the ionosorption and sulfuration-desulfuration models, as mentioned above, present a clear mechanism for sensing H_2_S gas by sensors based on the ZnO nanostructure.

The H_2_S gas-sensing mechanism of the sensor based on the ZnO/CuO-H structure differs from the sensor based on pure ZnO in that it is related to the formation of a heterojunction between p-type CuO and n-type ZnO semiconductors. The formation of p-n heterojunctions at the interface between CuO and ZnO was demonstrated by analyzing the electrical properties of the samples, as mentioned in the previous section. The response of H_2_S in the sensor based on the CuO-decorated ZnO hierarchical structure is attributed to three effects: (1) removability of the absorbed oxygen species caused by H_2_S as shown in Eq. (2) (for both CuO and ZnO), (2) sulfuration of ZnO and (3) with the subsequent generation of copper sulfide due to the reaction of CuO with the H_2_S target gas. The former two effects were discussed above. The third effect is considered next. Copper sulfides are considered to be p-type semiconductors because of the presence of cationic vacancies in the lattice structure. However, copper (II) sulfide (CuS) is unstable, and can be transformed into copper (I) sulfide (Cu_2_S) at high temperatures (>103 °C)^45^,^46^. Therefore, the formation of copper sulfide in the Cu_2_S structure caused by the reaction between CuO and H_2_S via


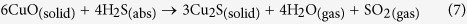


in the measured working temperature range (150 → 300 °C) is more dominant than that of CuS. The high electrical conductivity property of Cu_2_S was attributed to the short Cu-Cu distances, which are comparable with the Cu-S distances and resemble metallic Cu-Cu bonding. In addition, the specific resistivity of Cu_2_S is known to be around 4×10^−2^ Ωcm at 127 °C, and continues to decline at higher temperatures^47^,^48^. Therefore, the transition from CuO to Cu_2_S upon exposure to H_2_S causes a significant decrease of the potential barrier in the p-n junction of the sensor due to the change in the energy band structure, thereby increasing the conductivity of the sensor.

However, before the formation of Cu_2_S by Eq. (7), CuO nanoparticles are also shown to respond to H_2_S gas by displaying the behavior of a p-type oxide semiconductor sensor. Herein, we examine the H_2_S gas-sensing property of CuO materials based on CuO nanowires as mentioned before. In detail, the absorption of oxygen molecules in ambient air by Eq. (1) traps electrons from the valence band and hence increases the concentration of holes in CuO. When H_2_S gas is introduced, the H_2_S molecules react with the ionosorbed oxygen species according to Eq. (2). The electrons from the surface states are re-injected into the CuO semiconductor and recombined with the holes in the valence band resulting in a reduced concentration of holes (increasing the sensor resistance) as indicated by the CuO nanowires (average diameter of ~125 nm) sensor in Fig. S3b (black curve). Therefore, the reduction in the concentration of holes in the CuO nanoparticles under ambient H_2_S gas, as mentioned above, causes a decrease in surface conduction of CuO, thereby leading to increasing sensor resistance. However, for CuO nanowires with a smaller average diameter (~40 nm), the response of the sensor shows two steps (region I and II), as shown in Fig. S3b (red curve). In the first step (region I with an H_2_S gas injecting time of ~2 min), the sensor resistance increases upon exposure to 5 ppm H_2_S gas concentration. The increasing resistance of the sensor is attributed to the surface reaction between H_2_S and absorbed oxygen ions as expressed by Eq. (2), which causes a reduction in the concentration of holes in the CuO nanowires. Moreover, the formation of Cu_2_S by Eq. (7) occurs simultaneously with the surface reactions. However, the formation of Cu_2_S on CuO nanowires is not continuous. When the H_2_S gas injecting time is sufficiently long, the Cu_2_S structure is formed to an extent that it becomes a connecting bridge for electrical conductance. Therefore, the sensor resistance in the second step (region II) decreases by approximately three orders of magnitude compared to the baseline resistance after an H_2_S gas injecting time of 11 min, and this reduction is ascribed to the high conductivity of Cu_2_S. These observations indicate that the surface reactions and the sulfuration of CuO occur simultaneously during the initial response of the H_2_S gas, and that the sulfuration of CuO is eventually more dominant. After the supply of H_2_S gas is discontinued, the sensor recovers slowly to its initial state (Fig. S3b) because of the re-oxidation of Cu_2_S to CuO via


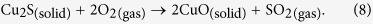


In the ZnO/CuO-H structure sensor, the CuO nanoparticles have a small diameter (tens of nanometers) as mentioned in the previous section (Fig. 2d-inset) suggesting that its surface becomes more active. Therefore, the rapid conversion from CuO into Cu_2_S upon exposure to H_2_S gas by Eq. (7) was considered the main factor for the improvement in the sensitivity of the ZnO/CuO-H sensor. The formation of highly conducting Cu_2_S nanoparticles from CuO in ambient H_2_S on the ZnO surface significantly reduces the contact barrier at the interface between CuO and ZnO, thereby resulting in an increase in the conductivity of the sensor. The curves that were determined for the response and recovery times based on Eqs (3) and (4) deviated because first-order surface reaction kinetics were assumed as shown in Fig. S4c,d. Nevertheless, the above definition was used to examine the response and recovery times for ZnO/CuO sensors as shown in Fig. S5. Longer response times and shorter recovery times were obtained for the ZnO/CuO-H sensor compared to the ZnO-H sensor.

The above results show that the sensitivity of the ZnO-H structure sensor was improved after decoration with CuO nanoparticles. Thus, the effect of the amount of CuO was also investigated and analyzed in this work. This was done by varying the amount of CuO by controlling the Cu salt concentration from 0 mM to 40 mM during deposition. The amount of decorated CuO was analyzed by conducting XPS measurements. In this way we found that the amount of decorated CuO in the ZnO/CuO-H structure increased as the Cu salt concentration was increased. The atomic ratios of Cu to Zn (Cu/Zn ratios) were 3.95%, 7.59%, 19.11%, 33.46%, and 110.44% at different Cu salt concentrations of 2.5, 5, 10, 20, and 40 mM, respectively (Fig. 7d inset). The sensitivity of the sensor towards 5 ppm H_2_S in dry air at a working temperature of 200 °C is plotted as a function of the amount of CuO catalyst clusters in Fig. 7d. The sensitivity of the ZnO/CuO-H sensors is mostly higher than that of the pure ZnO-H sensor. The results show that the sensitivity of the sensors increases with an increasing amount of CuO and reaches its maximum value at a Cu salt concentration of approximately 5 mM (Cu/Zn ratio of 7.59%) for deposition. However, the sensitivity of the sensor was reduced at higher CuO concentrations. As mentioned above, the formation of p-n junctions occurred as a result of the decoration of the ZnO nanostructure with CuO. Therefore, the sensitivity of the ZnO/CuO-H sensor improved with increasing CuO concentration due to the increasing number of p-n junctions on the ZnO surface. However, high CuO coverage of the ZnO surface only leads to a partial conversion of CuO to Cu_2_S, and a large amount of p-type semiconductor CuO remains. These effects may cause a decline in sensitivity upon high CuO concentrations. The competition between these factors shows an optimum Cu salt concentration of 5 mM for CuO decoration.

**Figure 8:**
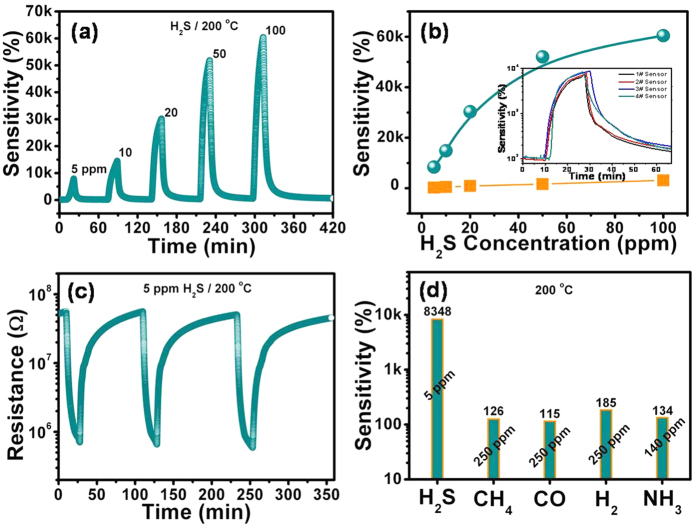
Sensitivity of ZnO/CuO-H (5 mM) sensor to various H_2_S gas concentrations at optimal working temperature of 200 °C (**a**) and its summary in sensing H_2_S concentration (**b**). Reproducibility checked for ZnO/CuO-H (5 mM) sensor upon exposure to 5 ppm H_2_S at optimal working temperature of 200 °C (**c**). Selectivity histogram of ZnO/CuO-H (5 mM) structure sensors towards different gases: H_2_S (5 ppm), CH_4_ (250 ppm), CO (250 ppm), H_2_ (250 ppm), and NH_3_ (140 ppm) at a working temperature of 200 °C. (**d**) Inset in (**b**) shows uniform sensitivity (to 5 ppm H_2_S) of four ZnO/CuO-H (5 mM) sensors that are selected randomly in fabricated process.

The response behavior of ZnO-H and ZnO/CuO-H (5 mM) (Fig. 8a) structure sensors to H_2_S was measured at various concentrations of 5, 10, 20, 50, and 100 ppm diluted in dry air at a working temperature of 200 °C. The sensitivity of these sensors at different H_2_S gas concentrations are also summarized in Fig. 8b. A sensitivity of 30300% was obtained with the ZnO/CuO-H (5 mM) sensor at a 20 ppm H_2_S gas concentration, which is much higher than the sensitivity of 907% obtained from the pure ZnO-H sensor. The catalytic effect of CuO nanoparticles can be realized for significant improvement at all measured H_2_S gas concentrations. The relationship between the sensitivity and H_2_S gas concentration was found to be linear for the pure ZnO-H sensor, whereas for the ZnO/CuO-H sensor this linearity was realized at low H_2_S concentrations (<20 ppm), and the sensitivity tends to saturation at high concentrations (Fig. 8b). These results can be explained by the near-perfect conversion of CuO to Cu_2_S. Although lower concentrations of H_2_S gas (<5 ppm) cannot be precisely prepared in our lab, the limit of detection (LOD) herein can be reasonably estimated to be around tens of ppb by assessing the noise-floor (*δ*) and the slope value (*s*) of the linear curve fitting of sensor sensitivity (%) versus the gas concentration (ppm) at a low concentration range (<20 ppm). The LOD value (=3*δ/s*) was estimated to be about 0.015 ppm (or 15 ppb) from the slope value of 1470 ± 60 and noise-floor of 7.65, suggesting that the present sensors can be used to detect H_2_S gas at ultralow concentrations down to the ppb level. The inset in Fig. 8b shows the sensitivity of the sensor upon exposure to 5 ppm H_2_S gas of four CuO (5 mM) decorated ZnO sensors that were selected randomly inform the fabrication process. It is clear that the sensitivity levels of these sensors are very similar, which indicates the expected uniformity of the sensors. The reproducibility of the optimum ZnO/CuO-H (5 mM) structure sensor at a working temperature of 200 °C upon exposure to a concentration of 5 ppm H_2_S diluted in dry air is examined in Fig. 8c. In addition, the ZnO/CuO-H (5 mM) sensor also showed excellent selectivity to H_2_S gas, as is evident from Fig. 8d.

## Conclusions

H_2_S sensors based on an open-space porous CuO-decorated ZnO hierarchical structure were developed. The ZnO-H structures were fabricated by the electrospinning method, followed by hydrothermal growth and thermal treatment. The CuO nanoparticles were then deposited onto the ZnO-H structures using a wet method. H_2_S gas reactions were detected on both pure ZnO-H and ZnO/CuO-H surfaces. The CuO-decorated ZnO-H sensor exhibited a significant improvement in its H_2_S sensing performance because of the formation of p-CuO/n-ZnO junctions. The optimal sensor structure was determined to be a ZnO/CuO-H (5 mM) structure. The H_2_S gas response and recovery mechanism of sensors was also explained in detail in terms of the surface reactions and the sulfuration as well as desulfuration of ZnO and CuO upon exposure to H_2_S gas diluted by dry air. Moreover, another important function of CuO was the reduction of the working temperature. The optimization of the structure yielded a sensitivity of 8384% upon exposure to a concentration of 5 ppm H_2_S gas at a working temperature of 200 °C. This result is one of the best reported in the literature to date for sensors based on ZnO/CuO hybrid nanostructures. However, the reduction of the operating temperature and improvement of sensor performance constitute an immediate task for future studies on H_2_S sensors.

## Methods

### Materials

All chemicals were purchased from Sigma-Aldrich Co., Ltd and utilized without further purification: poly(vinylpyrrolidone) (PVP) (wt 360000), zinc acetatedihydrate (ZnAc) (Zn(CH_3_COO)_2_ ·2H_2_O, 99.99%), zinc nitratehexahydrate (Zn(NO_3_)_2_.6H_2_O, 98%), hexamethylenetetramine (HMTA) (C_2_H_12_N_4_, 99%), Copper (II) nitrate hemi(pentahydrate) (Cu(NO_3_)_2_.2.5H_2_O, 98%), distilled water (DI, 18.4 MΩ/cm), *N*,*N*-dimethylformamide (DMF) (HCON(CH_3_)_2_, ≥99%), and ethyl alcohol (C_2_H_5_OH).

### Preparation of ZnO Hierarchical Structures on Al_2_O_3_ substrate

ZnAc – PVP nanofibers were electrospun from a solution of DMF (9.5 mL), PVP (0.8 g) and ZnAc (0.8 M). DMF was used as a solvent to dissolve PVP and ZnAc. A mixture of DMF and PVP was stirred for 6 h, after which ZnAc was added and stirred for an additional 4 h. In the electrospinning process, the solution was injected through a stainless steel needle (30 gauge, orifice diameter of 140 μm) that connected to a high voltage DC power supply of 26 kV across a distance of 15 cm towards the grounded collector. The solution was continuously injected by a syringe pump at a rate of 0.6 mL/h. The Au patterned Al_2_O_3_ substrates (2.5 × 2.5 mm), part of which (1.5 × 2.5 mm) was fixed by using tape (Fig. 1c), were placed on a grounded collector for the accumulation of nanofibers. Later, the ZnAc-PVP composite nanofibers were oxidized at 500 °C (at a heating rate of 15 °C/min) in an ambient atmospheric environment for 3 h to remove the PVP and form the ZnO-Fs.

For the hydrothermal synthesis of the ZnO-H structure, a mixture of 0.04 M of aqueous solution that included Zn(NO_3_)_2_.6H_2_O and C_6_H_12_N_4_ (1:1 ratio) was prepared based on a previous study^49^,^50^. The ZnO-Fs containing Al_2_O_3_ substrates were placed in this solution at 90 °C for 4 h to allow the ZnO nanorods to grow around the nanofibers. After the formation of the ZnO-H structures, the samples were annealed in air at 500 °C for 2 h for stability of ZnO structures.

### Deposition of CuO nanoparticles on ZnO hierarchical structures

CuO nanoparticles were deposited on the surface of ZnO-H structures using a wet method. Copper salt solutions were prepared by dissolving different amounts of Cu(NO_3_)_2_.2.5H_2_O salt in ethanol solvent at room temperature. The above ZnO-H samples were dip-coated with CuO by immersing in copper salt solutions of 0, 2.5, 5, 10, 20, and 40 mM of Cu(NO_3_)_2_.2.5H_2_O under UV (365 nm) illumination at 6.3 μW/cm^2^ for 1 min. Oxidation of the samples at 350 °C for 30 min resulted in the formation of CuO nanoparticles on ZnO-H structures. Figure 1 shows a flowchart of the fabrication process for open space porous CuO-decorated ZnO hierarchical nanostructures and a schematic diagram of the gas-sensing apparatus.

### Characterization and gas-sensing measurement

The surface morphology of the hierarchical structures was investigated by field emission scanning electron microscopy (FE-SEM, MIRA II LMH, Tescan, USA). The structural and optical properties were investigated by X-ray diffraction (XRD Panalytical, Netherlands) using Cu Kα radiation with a Ni filter, X-ray photoelectron spectroscopy (XPS; VG Multilab 2000; ThermoVG Scientific, UK), UV-vis-NIR spectroscopy (Jasco V670, Japan), and photoluminescence measurements using an FP-6500 spectrofluorometer (JASCO, Tokyo, Japan) using an excited wavelength of 325 nm. Resistance measurements and gas-sensing properties were measured using a pico-ammeter/voltage source (Keithley 6487). We used 1000 ppm H_2_S gas diluted in nitrogen as the analyte gas. The gas was further diluted in dry air by varying the concentration of H_2_S gas at a constant dry air flow rate of 300 sccm when fed into the test chamber as previously reported^5^,^51^.

Before each gas-sensing measurement, the sensors were preheated at the highest testing temperature to stabilize the layer of sensing material, and then reduced to the desired working temperature. Before each measurement of the gas sensing property at the given working temperature, the current-voltage (I-V) property of the sensor was measured by varying the applied voltage from −2 to 2 V to determine the contact property between the sensing material and Au electrodes. For gas sensing measurements, the applied dc voltage was fixed at a specific value of 1 V and the change of current (or resistance) of the sensor versus time was recorded under a continuous flow of gas with a constant flow rate (*q*_*t*_ = 300 sccm) of dry air. The gas-sensing properties were measured by diluting the target gases of H_2_S, NH_3_, CO, CH_4_, and H_2_ with their initial concentration in N_2_ of 1000 ppm in dry air by using a mass flow controller (MFC) with a flow rate of q_c_ (sccm) to various desired concentrations before loading into the chamber.

If the target gas was diluted by N_2_ at an initial concentration of C_o_ (ppm), the relative concentration (C_t_ (ppm)) of target gas in the gas mixture is calculated by:


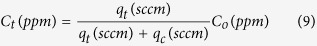


## Additional Information

**How to cite this article**: Vuong, N. M. *et al.* CuO-Decorated ZnO Hierarchical Nanostructures as Efficient and Established Sensing Materials for H_2_S Gas Sensors. *Sci. Rep.* **6**, 26736; doi: 10.1038/srep26736 (2016).

## Supplementary Material

Supplementary Information

## Figures and Tables

**Table 1:** CuO-decorated ZnO heterojunction sensors reported to detect H_2_S gas molecules.

Year	Morphology	Concentration (ppm)	S (R_a_/R_g_)	Temperature (°C)	S (%)/1 ppm	τ_Res_/τ_Rec_ (s)	Ref.
2003	Powder	50	20	108	40	13/5	16
2012	Hollow sphere	5	32.5	336	650	47/–(a)	11
2012	Nanowire	5	28	200	560	360/1800(a)	17
2012	Nanorod	100	39	100	39	120/>120(a)	14
2012	Nanorod	50	~889	500	1778	950/50(a)	15
2013	Flower	100	25	220	25	–/–	12
2015	Hierarchical	5	83.5	200	1670	572/65	[this study]

^(a)^The response time is defined as the time required for reaching 90% of the full response change of the sensor after the testing gas enters and the recovery time is defined as the time taken to fall to 10% of its maximum response after the testing gas exits.
