# Supplementary material for: CuO-Decorated ZnO Hierarchical Nanostructures as Efficient and Established Sensing Materials for H2S Gas Sensors
Source: Sci Rep. 2016 May 27;6:26736. doi: 10.1038/srep26736 (PMC4882619; doi:10.1038/srep26736)
Supplement: Supplementary Information [file srep26736-s1.doc]

Supplementary Materials:

CuO-Decorated ZnO Hierarchical Nanostructures as Efficient and Established Sensing Materials for H_2_S Gas Sensors

Nguyen Minh Vuong,^1,2^ Nguyen Duc Chinh,^3^ Bui The Huy,^1^ Yong-Ill Lee^*,1^

^1^Department of Chemistry, Changwon National University, Changwon 641-773, Republic of Korea

^2^Department of Physics, Quy Nhon University, 170 An Duong Vuong, Quy Nhon, Binh Dinh, Vietnam

^3^Department of Materials Science and Engineering, Chungnam National University, Daejeon, 305-764 Republic of Korea

**Corresponding authors:** *Yong-Ill Lee: [yilee@changwon.ac.kr](mailto:yilee@changwon.ac.kr)

**1. Photoluminescence spectrum of ZnO-H structure**


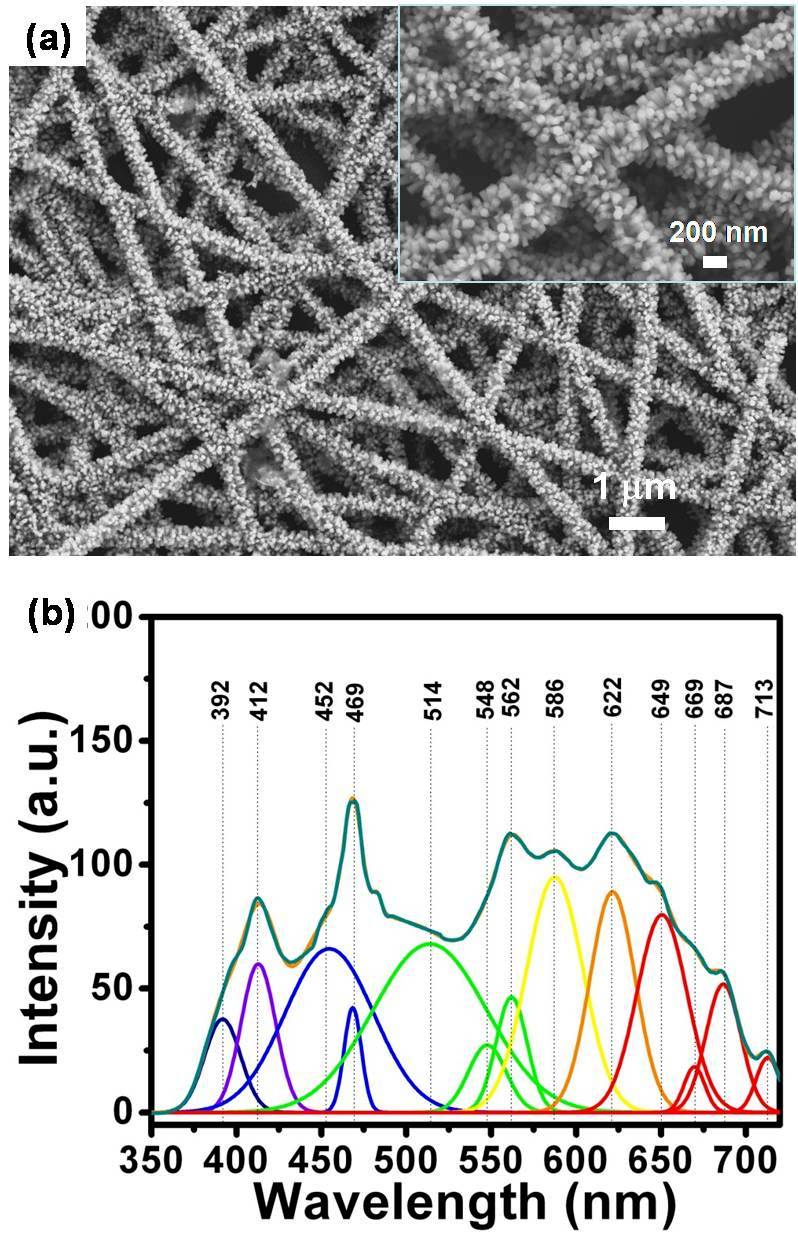


**Figure S1**. SEM image (a) and Gaussian fitted curves of emission bands (b) of the ZnO-H nanostructure prepared on a glass substrate

**2. Morphology and structural properties of CuO nanowire structure**


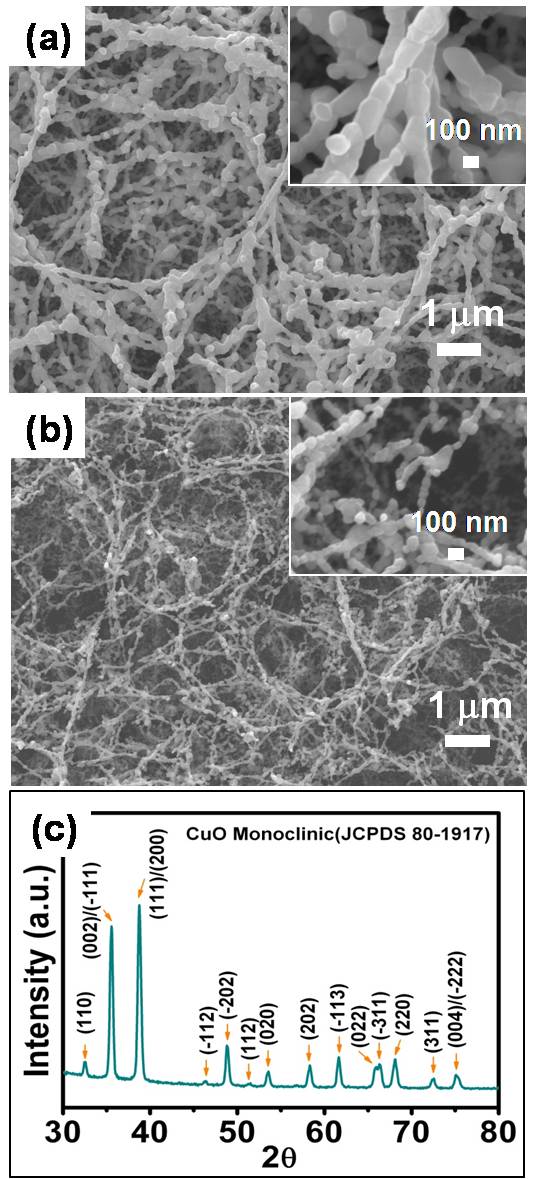


**Figure S2**. SEM images of CuO nanowire structures with deposition times of 8 min (a) and 2 min (b). Inset images show high-magnification SEM. XRD pattern of CuO (8 min) nanowire structure.

**3. Electrical and H_2_S gas-sensing properties of CuO nanowire structures**


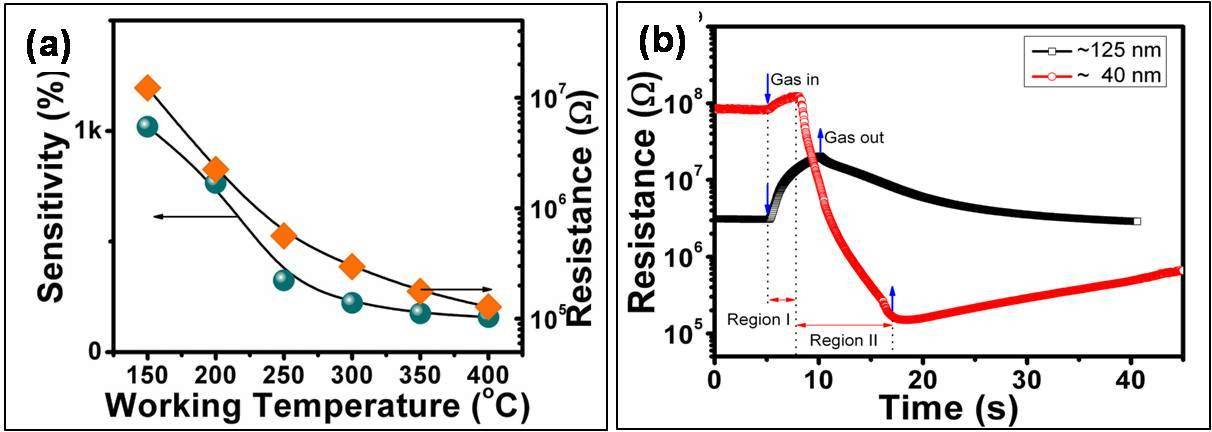


**Figure S3**. (a) Dependence of the resistance and the sensitivity (R_f_/R_i_) towards 5 ppm H_2_S gas of CuO (8 min) nanowire sensor on different working temperatures. (b) Gas-sensing properties of CuO nanowires with the deposition times of 8 min (black curve) and 2 min (red curve) upon exposure to 5 ppm H_2_S gas at working temperature of 200 °C.

**4. Response and recovery properties of ZnO-H and ZnO/CuO-H structures**


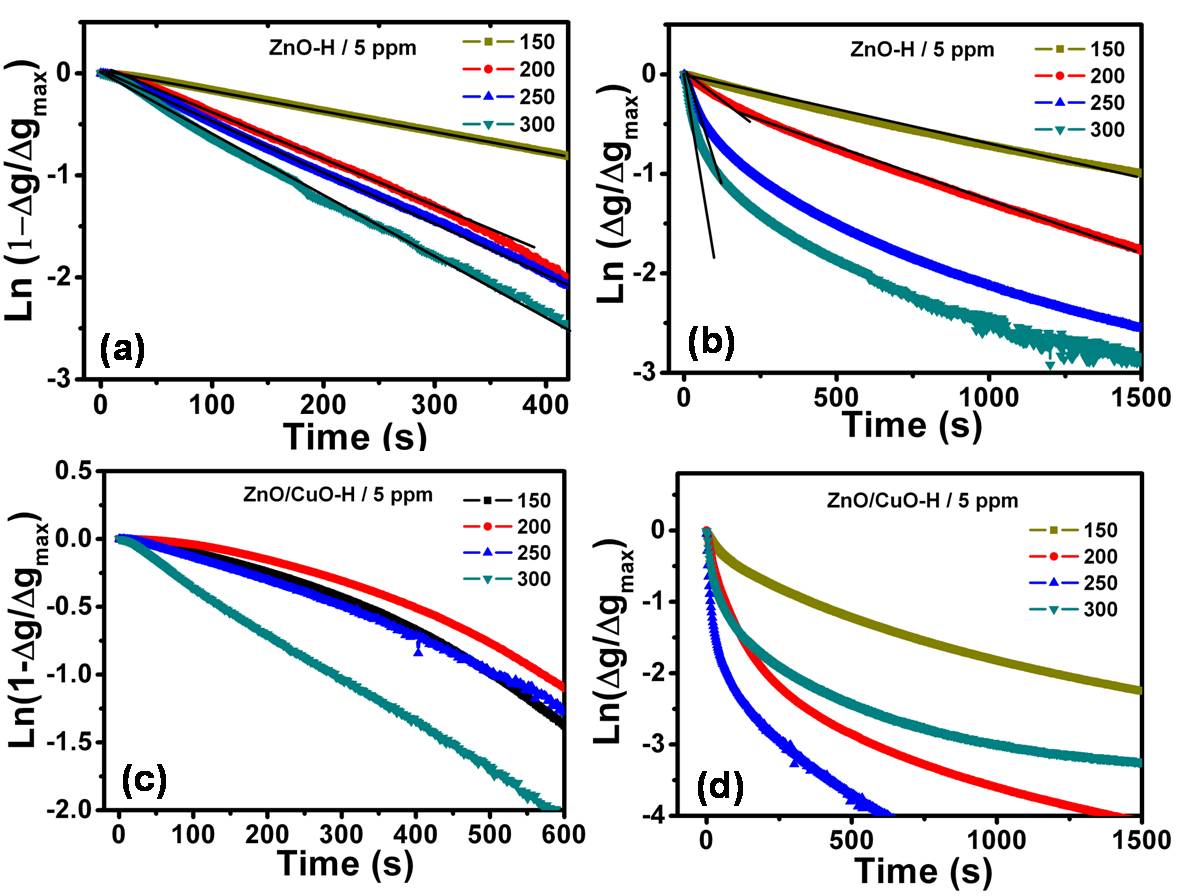


**Figure S4**. Logarithmic dependence of (a) (1 – *Δg/Δg_m_*_ax_) and (b) *Δg/Δg_max_* on the time to obtain the reaction time constants at different working temperatures of the ZnO-H structure sensor. Logarithmic dependence of (c) (1 – *Δg/Δg_m_*_ax_) and (d) *Δg/Δg_max_* on the time to obtain the reaction time constants at different working temperatures of the ZnO/CuO-H structure sensor.


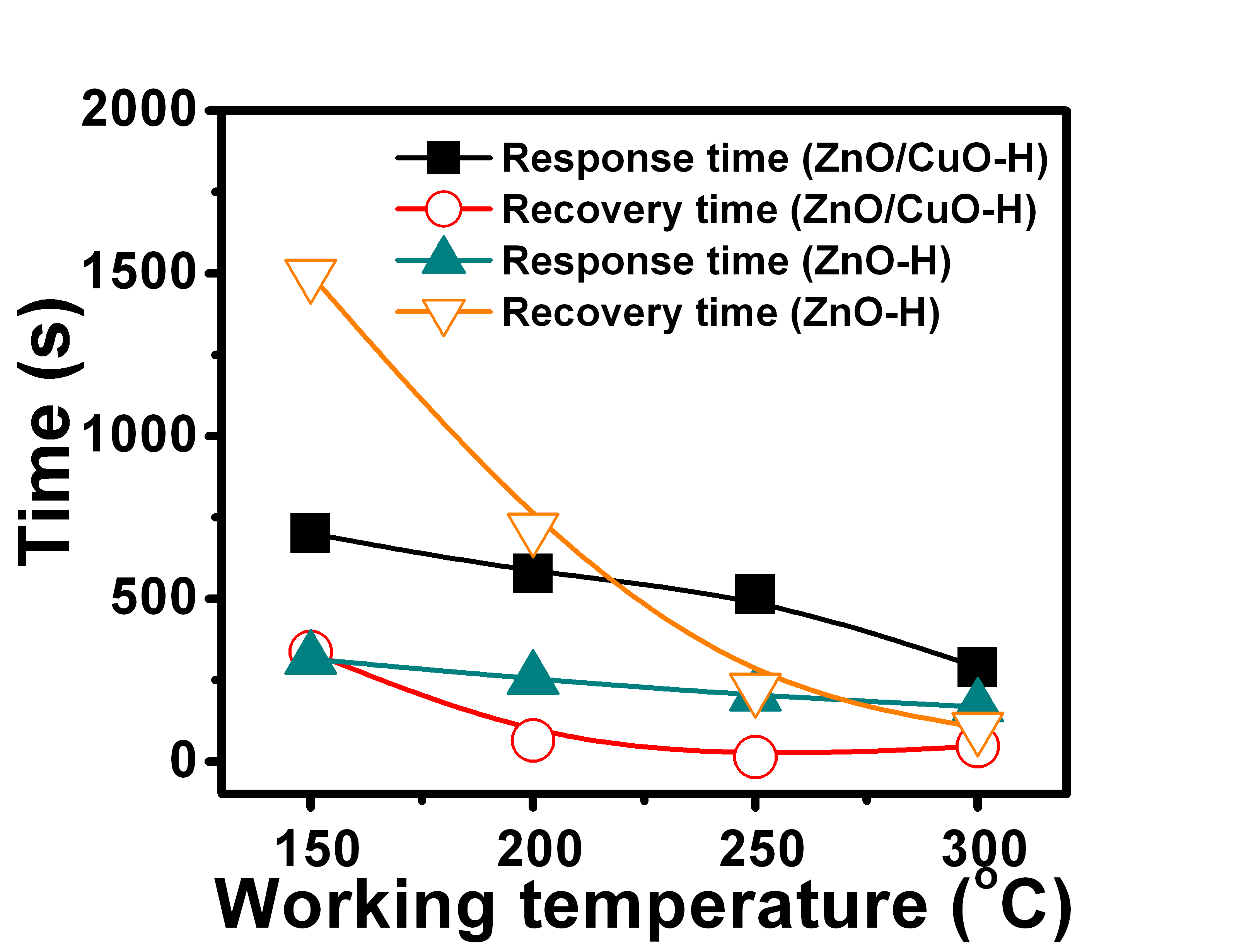


**Figure S5**. Dependence of the response and recovery times on different working temperatures of sensors based on ZnO-H and ZnO/CuO-H (5 mM) structures upon exposure to 5 ppm H_2_S.
